# How participatory arts can contribute to Dutch older adults’ wellbeing – revisiting a taxonomy of arts interventions for people with dementia

**DOI:** 10.1080/17533015.2022.2035417

**Published:** 2022-02-15

**Authors:** Yosheng Liu, Barbara Groot, Lieke de Kock, Tineke Abma, Christine Dedding

**Affiliations:** a Department of Ethics, Law and Humanities, Amsterdam Umc, Amsterdam, The Netherlands; b Leyden Academy on Vitality and Aging, Leiden, The Netherlands; c Department Public Health and Primary Care, Leiden University Medical Centre, Leiden University, Leiden, The Netherlands

**Keywords:** Older adults, participatory arts, wellbeing, underlying mechanisms, taxonomy of art interventions

## Abstract

**Background:**

A growing body of evidence suggests the positive impact of arts on health and wellbeing. The mechanisms underlying the impact however, remain overlooked.

**Methods:**

38 Semi-structured interviews were held with 30 older adults and 10 artists, involved in five participatory art projects in the Netherlands. Case-based framework and cross-over analyses were done on the basis of Cousins et al.’s taxonomy.

**Results:**

Participatory art initiatives contributed to the wellbeing of older adults in a complex interplay with the artist, art form, group of participants, material aspects and continuity of activities. A welcoming environment appeared a consistent underlying mechanism for participants to grow on a personal and artistic level, connect with others and feel supported in their psychosocial wellbeing.

**Conclusion:**

This article demonstrates the important social function participatory art can have for older adults, and argues for the importance of a thorough consideration of the context wherein underlying mechanisms and outcomes emerge.

## Introduction

A rapidly increasing aging population challenges global and public health ideals, scholars and practitioners in promoting and ensuring health and wellbeing in a later stage of the life course (Fancourt & Finn, 2019). Aging increases the chance of vulnerability, loneliness and lack of meaning in life, posing significant challenges to national healthcare systems and social care services (All-Party Parliamentary Group on Arts Health & Wellbeing, 2017; Van Campen, 2020). One promising strategy for addressing these challenges is participatory arts activities. In a global synthesis on arts and health research, the WHO outlined the potential beneficial role for arts in healthcare and public health domains for health promotion, prevention, management and treatment (Fancourt & Finn, 2019). This outline is based on a growing body of evidence signifying positive physical, mental and social health and wellbeing effects among various age groups when they engage in art and cultural activities. Other authors also point to the well-established link between participatory art activities and health and wellbeing (Clift, 2020; Curtis et al., 2018; Fancourt & Finn, 2019; Fraser et al., 2015; Gordon-Nesbitt & Howarth, 2020; Gray, 2008).

This growing international arts and health field is characterized by a dominant focus on outcomes, effects and impact studies while the mechanisms that underlie the effects of art activities often remain understudied (i.a. Bradfield, 2020; Daykin, 2019; Dunphy et al., 2019; Matarasso, 2019; Wakeling, 2014). Regarding terminology, processes and mechanisms through which benefits of art activities may occur, seem to be used interchangeably. Cousins and colleagues (Cousins et al., 2020, p. 125) describe mechanisms as “hidden processes,” whereas Windle et al., (2018, pp. 703–704) refers to “how contexts (by whom and how)” trigger “theoretical mechanisms (why) through which any benefits of arts activities may occur”. Daykin et al., (2021, p. 138) rather conceptualizes processes as “emotions, responses, actions and behaviors that lead to changes in wellbeing”, while Dunphy et al., (2019, p. 8) define processes as “seen to elicit change in the client”, and mechanisms as “what occurs within the client that results in change”. Despite this lacking consensus, all concepts come down to questioning how and why art activities produce their effects – instead of what the impact is. We continue by referring to underlying mechanisms when addressing these how and why questions.

Only a few researchers explicitly explored these mechanisms. Fancourt et al. (2014) reviewed the psycho-neuro-immunological effects of music and referred to a limited discussion on potential underlying mechanisms. Others have theorized on neural, hormonal, and psychological mechanisms that underlie the effect of music on social cohesion and post-stroke rehabilitation (Särkämö & Soto, 2012; Tarr et al., 2014). In that regard, Daykin et al. (2021) argued how a focus on biological, physiological, hormonal and neurological pathways, reflects a certain level of medicalization into this field. Dunphy et al. (2019) reviewed the mechanisms of creative arts interventions in relation to depression amongst older adults, and also concluded a lack of (substantiated) attending to the topic in the field of psychology. Together, these attempts disclose the current theoretical stage of research into mechanisms that underlie the impact, and many scholars recognize the need for a common conceptual framework for guiding research and art practices (Bradfield, 2020; Cousins et al., 2020; Dileo & Bradt, 2009; Tay et al., 2018; Windle et al., 2018). The suggested conceptual frameworks that transcend medical pathways and adopt a more holistic approach to art and health practices – Cousins et al., 2019), Tay et al., (2018); Windle et al., (2018) – however, remain relatively new, conceptual, and unapplied in research (Daykin, 2019; Matarasso, 2019).

Our aim is twofold. Firstly, this study investigates the mechanisms that underlie potential benefits of active art engagement in terms of older adults’ wellbeing. We do this through the lens of Cousins et al.,’s (2020) taxonomy of arts interventions for people with dementia. An epistemological rationale for adopting this taxonomy is its underpinning by a realist methodology, which incorporates real-world expertise to explore “what works, for whom, under what circumstances and why” (Wong et al.,, 2013, p. 2). To investigate how art activities work, Cousins et al., (2020) suggested the context-mechanism-outcome (CMO) configuration as a realist theory. Similarly, other authors presented this theory (cf., Carswell et al., 2021; Windle et al., 2014; Wong et al., 2013) as an analytic tool for focusing on context (C), mechanisms (M) and outcomes (O) in complex interventions.

Secondly, our aim is to validate the claim made by Cousins et al., (2020) that the Principles dimension (Table 1) – fundamental to the taxonomy’s development (Cousins et al., 2019) – is observable and applicable to multiple art forms in different care settings, and people at various stages of dementia. This is important, because the empirical underpinning of Cousins et al., (2020) taxonomy is mainly based on music therapy in care institutions, for people with dementia. Next to validating, we go one step further by including a more general group of older adults. This seems especially relevant in the context of creative aging. Closely related to the arts and health field, creative aging entails “creative engagement in later life” and covers a range of practices wherein “older adults engage in art programs, with a specific focus on social engagement and developing creative skills” (Bradfield, 2020, p. 93). Although often associated with loss, we can complement our understanding of ageing as “a period of personal growth, creativity and productivity” (World Health Assembly, 69, 2016, p. 16). In his article, Stern (1967, p. 62) argues that “creative aging begins now – if we wish to be creative when we are fortunate enough to be aged.” In relation to aging populations and increasing life expectancies, we highlight the importance to start unpacking how participatory arts can possibly contribute to meaningful and creative later stages of life – early onwards, from 50 years of age and over, and regardless of (dis)abilities.

**Table 1.** Principles dimension, Cousins et al. (2020).

Principle description	Principle summary
**Involvement**: *Arts interventions involve a range of participants, are welcoming, mutual, and equalizing, and include limitless activities that can embed the arts into everyday life.*	*The arts can include and welcome people.*
**Connection**: *Arts interventions can facilitate connection to the self and others through bonding, morale, and reminiscence, enabling social interaction and relationship building.*	*The arts can enable social interaction.*
**Expression**: *Arts interventions allow participants to share and frame their emotions, sometimes without language, generating enjoyment, discussion, or challenge to make meaning.*	*The arts can help people share and process their emotions.*
**Transformation**: *Arts interventions might transform participants using creativity, imagination, and flow – transporting them to a different time, emotional space, or into the moment*	*The arts can transform people and change how they feel.*
**Engagement**: *Arts interventions engage and stimulate, possibly using improvisation or play, and give participants an opportunity to take part in a live activity.*	*The arts can be lively and fun.*
**Possibility**: *Arts interventions can allow failure-free achievement and the use or development of skills, encouraging curiosity in different places, and providing a focus on enrichment and potential*	*The arts can give people new experiences.*
**Selfhood**: *Arts interventions can generate purpose and feelings of independence, identity, and empowerment, while being personalized and accessible to individual needs and choices.*	*The arts can create a sense of identity.*
**Humanity**: *Arts interventions can offer respite by validating, regulating, or stabilizing mood, and enabling a process of trust, relaxation, and hope.*	*The arts can be relaxing and supportive.*

## Methods

### Methodology

In this study, we focused on the lived experiences of older adults and artists, by employing qualitative methods that are underpinned by a realist methodology. In its approach to social reality, realism positions itself in between positivism (“observable external reality out there”) and constructivism (“reality is subject to interpretative understanding”; Wong et al., 2013). Realist inquiry wields explanatory strategies by investigating causal relationships between contexts, mechanisms and outcomes of social interventions (Pawson et al., 2005). Therein, it takes a generative conception of causality. Rather than looking for associated variables, like successionist views, realists seek to understand how associations emerge in the first place. Generative/underlying mechanisms thus account for those processes that allow certain outcomes to emerge; they both constitute and are outcomes (Pawson & Tilley, 1997, p. 4).

### Study design

We employed a multiple case study design that is embedded within a more extensive participatory action research study in the Netherlands (Groot et al., 2021), in order to explore the relationships between outcomes and mechanisms in different contexts. Stake (2013) argues that multiple case studies ideally incorporate in between four and 10 cases in order to balance case interactions with intelligibility. In accordance, we purposefully selected five art projects as single cases, out of 19 initiatives in the wider study. Each case’s art activities were led by at least one artist, who’s disciplinary background varied from dance, theater and visual arts to journalism, freelancing and media production. The amount of participants per case-activities varied from five to 45. Participants were in between 50 and 104 years of age and had diverse physical and mental care needs. Together, the cases included multiple art forms such as dance, singing and visual arts. All art activities focused on the active engagement of participants and included aesthetic, personal and social rather than therapeutic aims. Each case operated in an urban context and activity settings ranged from nursing homes to community centers, museums, churches, chapels, public spaces, city parks and online platforms. Apart from cases B and C which are project based, all activities were continuous throughout the year (Table 2).

**Table 2.** Description of the included art initiatives and participants.

Art-form	Description of art activities and participants
*Dance*	Dance lessons and events in communities and care facilities; older adults ranged from independent of care, to various stages of dementia and physical frailty (age: 60–100 years; n = 150 in total organization; n = 5–20 in group lessons) Dance labs working towards performances for older adults in care facilities; older adults were both dependent and independent of care (age: 60–99 years; n = 20–25)
*Choir singing*	Choir with residents of an elderly care facility working towards a performance; older adults ranged from independent of care to various stages of dementia (age: 50–104 years; n = 45)
*Visual art*	Weekly walk-in intergenerational studio in the community; older adults suffered from various psychosocial issues: psychological trauma, loneliness, discrimination (age: 50–90; n = 10) Weekly walk-in intergenerational meetings focusing on drawing; older adults suffered from a range of psychosocial problems: drug addiction, loneliness, burn-out, depression (age: 50–99; n = 15–20)

### Data collection

We discern three data collection cycles in 12 months (February 2020 – February 2021). In cycle one (February 2020 – April 2020), five face-to-face semi-structured interviews were conducted with eight artists on the context and specificity of art activities, their impact and potential underlying mechanisms (length: 45–80 minutes; 5 women-3 men). In cycle two (April 2020 – September 2021), 28 semi-structured telephone interviews were done with older adults (length: 30–80 minutes; age: 52–90; 19 women-9 men) on their lived experiences during art activities. In addition, 25 participant observations and informal interviews (3–15 minutes) where conducted during art activities, either live (n = 17) or online (n = 2) depending on COVID restrictive measures. In cycle three (October 2020 – February 2021), five respondent validation interviews were held (cf., Green & Thorogood, 2018). One was face-to-face with a male artist and two older male participants. The other interviews with seven artists were held online (length: 50–80 minutes; 5 women-2 men). All formal interviews were recorded and transcribed ad verbatim. Field notes were written down in a notebook.

### Data analysis

The data of the first cycle was analyzed thematically (cf., Braun & Clarke, 2012) with the help of data analysis software MAXQDA 2018. Identified themes informed the second cycle of data collection. Thereafter, case-based framework analyses (cf., Ritchie et al., 1994) were applied to the data of cycle one and two. Grounded in the perspectives of older adults and artists, five art projects were analyzed as separate cases based on the Principles dimension of Cousins et al., (2020). Their perspectives were triangulated with participant observations. The resulting analyses structured the respondent validation interviews. After validation, a cross-case analysis (cf., Stake, 2013) was conducted on the basis of the taxonomy. We applied charting, mapping and interpretation (cf., Ritchie et al., 1994) to all cases according to Cousins et al., (2020) Principles and Contextual dimensions. Herein, we wielded an instrumental approach to our multiple case study (cf., Stake, 2013) and focused primarily on commonalities and differences between cases rather than in-depth explorations of each case. Throughout all cycles, analyses were deliberated within the broader research team, consisting of researchers, older adult co-researchers and one artist.

### Ethics

**Figure 1.**
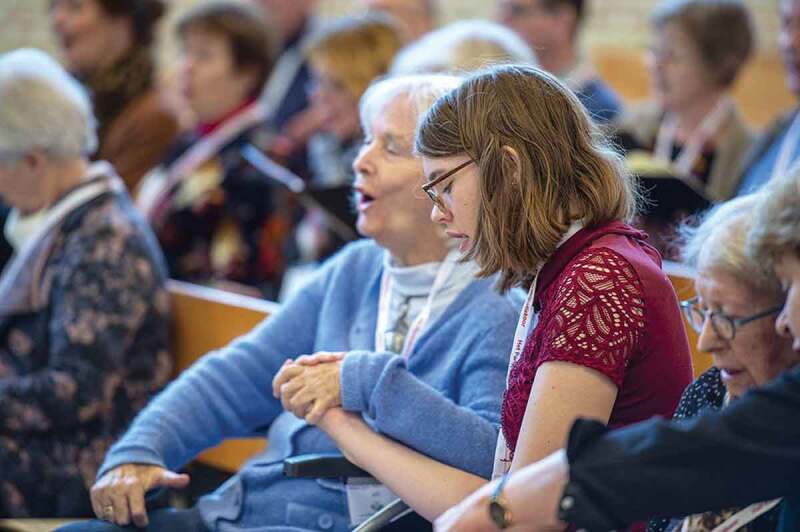
Older singer with dementia and her buddy singer, rehearsing in a chapel.

**Figure 2.**
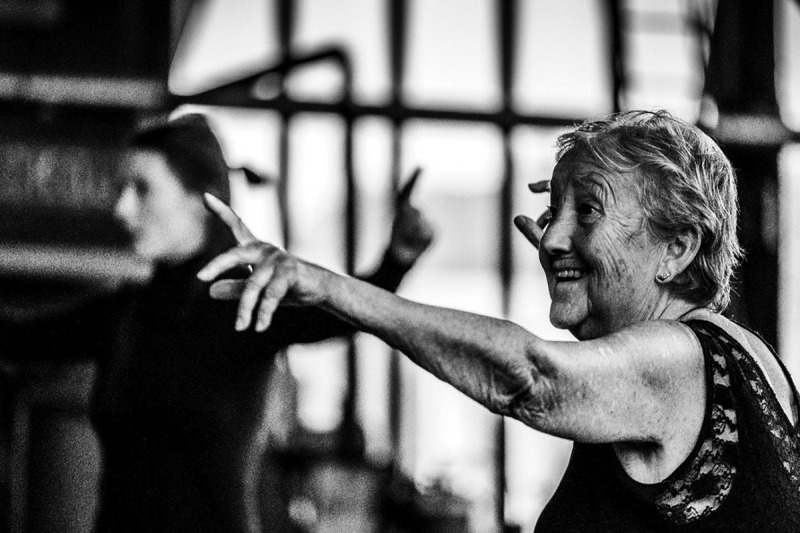
Older dancer during a dance workshop inside a museum.

This study was conducted according to the guidelines of the Declaration of Helsinki, and approved by the Medical Ethics Committee of Amsterdam UMC, location VUmc (IRB00002991) with number 2019.738 at 29–01-2020. Each participant gave verbal informed consent before interviews, entailing information about the research, handling the data and guaranteeing anonymity. Concerning Figures 1 and 2 (Figure Captions), all identifiable participants gave informed consent for the use of these images in this article.

Potographer: Zan van Alderwegen

Photographer: Lambert de Jong

## Results

In the following, we present five principles that were most prominent throughout the stories of older participants and artists: Connection, Possibility, Transformation, Humanity, and Involvement. Involvement appeared a consistent underlying mechanism in various principles. Therefore we present it throughout the results instead of separately.

### Connection: belonging, trust and freedom

Feeling part of an artistic community on which they could rely as a social resource was important for many participants. For some participants, especially in case of social isolation, artistic activities enabled participants to be around people. As one participant of a visual arts project explained:

It’s the only time in the week that I at all eat at the table with people. Because for the rest, I just eat at home you know, with my plate on my lap. So that is also a kind of home for me, or a very important event. And then we’re also talking about medical death and loneliness and other things that happen in the world, about hatred and envy and war, and refugees … And it doesn’t have to be, we also sometimes laugh really hard and have fun, that too.

(woman, 67 years, E)

Besides this social need, other participants mentioned that they could rely on their community in asking for practical help and advice, carpooling, or planning social activities together; helping and receiving in reciprocity. Having a common artistic goal, for example, in case of performances or exhibitions, facilitated bonding. As one member of a choir shared how engaging in group art gave a sense of connectedness: “It is so connecting … The music, being together. But also wanting to create something together. The intention. You all do it together … It was so harmonious.” (woman, 68 years, C). The artistic director explained how they put extra effort in facilitating social connections during singing rehearsals with bonding exercises, ice-breakers, and group conversations (Figure 1).

#### Involvement

An atmosphere of trust and acceptability was key for participants. “You are just accepted, and everyone is how they are.” (woman, 65 years, E) Such acceptance of identity was echoed by another participant who felt she could just be, free from social expectations:

If you don’t want to say anything all morning, you don’t say anything at all. Look you don’t have to! And I like that there. I’ve been in the care sector and my whole life it was: having to do something. If it wasn’t in my marriage it was at work, then it was at my other job, always “having to!” … Now I don’t have to do anything. I can! … Nothing is expected of me. I am coming. “Do you want coffee?” One time I say “No wait a minute.” I sit down and if I don’t want to do anything there for fifteen minutes, I just sit there.

(woman, 70 years, E)

In line with these experiences, the visual artist (man, E) emphasized how “new people pick [this sense of freedom] up quickly,” and how the material space facilitates this: “So here you don’t feel like you’re in a nursing home. This is not part of it. It’s messy. The atmosphere helps.” This demonstrates how besides common artistic goals, group (art) work, and the social role of artists, a welcoming environment enabled participants to make meaningful connections during workshops – an environment of a non-verbal and messy material quality, underpinned by experiences of trust, acceptance, and freedom. Enabling a focus on what is possible, increase in self-esteem and connections.

### Possibility: artistic and personal growth

Learning appeared central in the accounts of older adults as one participant illustrated her improvement in drawing:

I’ve really come a long way with what I’m doing. I became looser in what I do. Or it is getting easier, you know, it flows more. In the beginning I was really occupied with what I was, and what it looks like.

(woman, 67 years, E)

In a similar vein, another interviewee shared how she was “afraid to make mistakes” at the start: “My hands were shaking like this: Whooow!” (woman, 52 years, D) Reflecting on it, she concluded that making mistakes was crucial to a successful and interesting learning process. A dance participant felt challenged on a physical level:

It is especially nice to notice that you can do it with your aging body. Whereas, on videos you see all these young people ranting, and now we also learned how to drop from a twist to the ground, into the dive. It looks hip-fracture-dangerous! But then when you get the technique, and you turn into a spiral, yes, suddenly you succeed. That’s kicking! Especially to notice that that old body can do this, and is not left behind in broken bones.

(woman, 73 years, B)

With artistic guidance, she overcame initial apprehensiveness on her body’s capabilities; mastering “the dive” in the end was a positive experience. Other interviewees reverberated similar experiences in terms of increased self-esteem, artistic growth, and proud feelings: “I learned to draw. I’m not really an artist, but I couldn’t do anything before that! So when I do something I think: Wow! I admire myself when I see the result. So then I’m very happy!” (woman, 54 years, D). A dance participant argued learning to “explore possibilities and pushing boundaries” in daily situations. “I have the confidence that I can do more than my nose is long,” she continued, “And I really learned that through the art activity. This is what I mean by inner development. So that you are no longer so dependent.” (woman, 81 years, B)

A first condition for artistic learning and personal growth was experiencing a challenge. The different examples highlight the often personal and embodied nature of artistic challenges, and how they depend on individual life histories, activity, and art form. Making art can be new and scary for some at first. Other participants added how self-demanding attitudes and their need to perform perfectly challenged to self-reflect and accept artistic results. Artists deliberately stimulated artistic and personal unease, which ultimately led to new artistic and positive experiences:

She had made a drawing with flowers and said: “I’m not quite ready, what do I do?” I told her: “Make the background black.” And she always uses colors very carefully. So that black background was something strange to her, and she didn’t really know how to start. Then I started with black markers. She was shocked for a moment, but continued. And afterwards she said: “Wow, what a beautiful drawing that has become!” She showed her husband who said, “Well, she’s beautiful!” That kind of pride.

(man, visual artist, E)

A second important circumstance for growth is overcoming challenges. Besides mutual support from the group, the artist played an important supportive role, i.e. the dance choreographer deconstructing the duck into manageable movements. In aligning his dance instructions with the physical fitness of the group, the choreographer showed the importance of empathizing with participants’ life worlds, besides mastering the art form. One participant illustrated how contemporary dance and its focus on improvisation in a competition free environment, suited her physical fitness level:

It is just right for your movement- and body-level. Because it’s no longer possible to just roll on the ground and jump up. So you have a lot of limitations. And dance means exactly that you can do anything with your body. In fact you can do anything despite many limitations. And that’s just nice that there is a group that takes this seriously, and that you can continue to work at your own level. Without that technique, without there being a performance of how high you can go, or how fast …

(woman, 69 years, A)

Art can create conditions for overcoming challenges. Contemporary dance, with its freedom of movement, for example, enables participation throughout the life course regardless of physical fitness – as aging bodies might be accompanied by increasing disabilities. One dancer illustrated this vividly: “The amazing thing is when I dance I don’t suffer from anything! I think my body is completely set up for that. Very crazy. If I have to walk, my hip hurts after 500 m. Those movements are too one-sided.” (woman, 81 years, B). Similarly, in nursing homes and neighborhood centers, participants could participate freely from their (wheel)chairs in dance workshops. Furthermore, interviewees pointed to the importance of the group. A sense of “collective struggle” in a failure-free environment or the ability to exchange ideas and feedback contributed to overcoming challenges and collaborative and mutual learning.

A third aspect of growth was the ability to share artistic products with others during, for example, workshops, performances, or exhibitions. This art exposure contributed to participants’ self-esteem and sense of mastery: “You know your own value! And you’re proud of yourself. You think: I’m not weak yet, I can do it! You’re never too late to learn, if you want!” (woman, 52 years, D).

### Transformation: entering a state of flow in a non-judgmental environment

Many participants experienced “being in another world”, as a state of flow, with complete absorption into the activity wherein “we have actually forgotten what we can or cannot. Then, an inner source is flowing.” (woman, 70 years, B) As one interviewee shared her experiences during visual art workshops:

It’s like a door is closing, and the moment I’m there I just remember that moment. My mind is not going anywhere at that moment! I’m just occupied with my painting. At that moment it’s just my other … I’ve ended up in another world.

(woman, 54 years, D; Figure 2)

Artists played an important role in achieving flow. As one dance participant pointed to their artistic and intuitive competencies:

It’s a way of working. They talk very little, the two dance choreographers. It goes in a way that they initiate something and we continue. And then there are variations, it’s a kind of jam session. And every now and then they say: “Well go now with two or three, or just add this.” And like that you are all in a flow … They can do that very well, that you get into a creative flow. (woman, 70 years, B)

#### Involvement

Another woman commented: “I just forget to drink my coffee because I’m completely lost in what I’m making at the moment. But I don’t have to be ashamed of that, or that I’ll be addressed on it.” (woman, 67 years, E).

Interviewees shared how dance exercises on connecting resulted in the group taking precedence over individual performances; or how integrating live music with singing and various dance styles enabled everyone to contribute from their artistic strengths in a competition-free environment. The artistic director of the dance group explained how their artistic work is guided by specific social values:

Wherever we go, we listen to what there is, who we have in front of us, what the space gives. That listening also provides a feeling of security. To be, without expectations … And that people will start listening themselves. It’s about meeting each other in equality … And we respond to that … exercises are created for that, so that people end up in it.

(woman, B)

Altogether, this demonstrates how participants experienced a flow state through focusing on the art activity, connectedness, guidance of artists and a non-judgmental environment based on equality and listening.

### Humanity: flow in a relaxing space, supporting psychosocial wellbeing

In each art project, participants mentioned feeling good and relaxed afterward. Despite her high-stress levels due to psychological trauma, a participant shared: “From that first time … I experienced such peace … I’m just going there to relax, because I need that!” (woman, 54 years, D).

**Figure 3.**
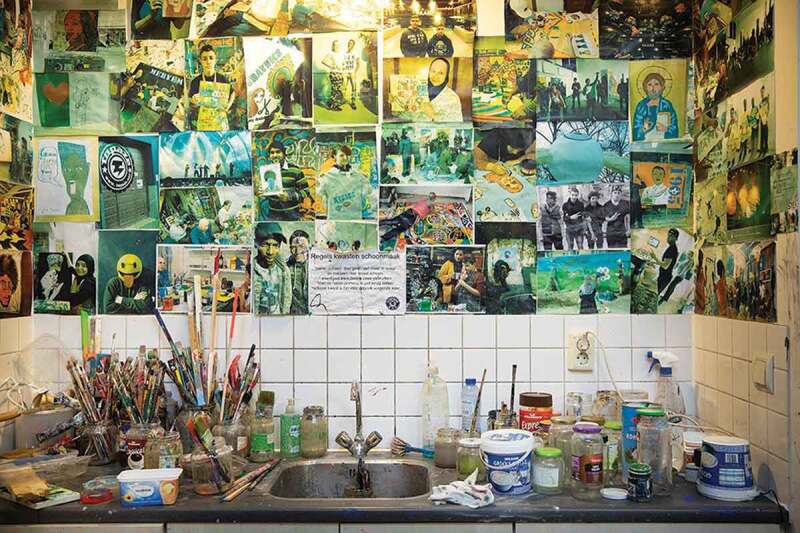
Art studio located in a church.

Some interviewees argued that experiencing flow alleviated emotional suffering: “It’s nice because that way you forget about the problems around you, you’re having a good time. Kind of liberating from your emotional and psychological problems … When I’m painting I forget the world. I am in my own world.” (woman, 65 years, D). One visual artist also emphasized the role of a different environment: “It’s a pretty safe place … for the people here. They come in, I don’t know what it is, maybe because it’s a church? … Even the biggest artist, they really relax here.” (woman, D; Figure 3)

Photographer: Janine Schrijver

Another interviewee elaborated on how the artists contributed to this relaxing effect:

They work together in a good team. You see the harmony. They support each other. And that also gives you a nice feeling, that you are in the right place! They get along well, and you also feel very relaxed … They really do it with love. Otherwise I wouldn’t have been here for three years!

(woman, 54 years, D)

Without reframing it into therapeutic terms, these stories disclose the potential for art activities to contribute to wellbeing. Several visual art participants echoed this. Participation aided to “restart” (man, 61, E) someone’s life after a burn-out, or supported another participant’s metamorphosis from “a dead bird into a tropical bird” (woman, 65 years, E). Another interviewee added: “Yes it is … therapy for life! … Through art and painting and doing other things I picked myself up little by little … With art you just go one step further, and one step further. You want to climb your way out.” (woman, 65 years, D). Underlying to this support, participants argued the importance of mutual recognition, emotional support from the group and artists, and continuity of activities. Daily art workshops offered one participant “important day structure. This became even more clear because of the COVID lockdown.” (man, 75, case D). One dancer added: “It really gives structure … Even that one time in two weeks gives a structure to time. You live up towards it, move away from it, you think about it, you live towards it again.” (woman, 76, case B).

## Discussion

Returning to the Context-Mechanism-Outcome-configuration (CMO), these results show the value of participatory arts for the psychosocial wellbeing of older adults (O). Participants felt supported by the empathic guidance of the artist and a stimulating and welcoming environment; they appreciated the opportunity to connect, learn, relax and get into a state of flow, thereby forgetting and/or working around physical or mental limitations.

When exploring underlying mechanisms (M), the entangled and fluid nature of principles becomes clear. Involvement operated as a facilitator for Connection, while Involvement and Connection contributed to experiences of Transformation. The latter principle on its turn, together with Involvement enabled Humanity, as one participant shared how being in her own artistic world made her forget emotional problems, and the artist added how the safety of the space added to psychosocial support. This discloses that outcomes and underlying mechanisms are intertwined and situated in a specific context (cf., Pawson & Tilley, 1997). Nevertheless, Involvement appeared a consistent underlying facilitator for other principles. This suggests older adults’ need for a warm, human and welcoming environment where they can connect and grow in the face of decreased social networks, increased loneliness, vulnerability, and lack of meaning (All-Party Parliamentary Group on Arts Health & Wellbeing, 2017; Van Campen, 2020). Other studies have also emphasized the importance of a welcoming environment. Camic et al., (2016) and Burnside et al., (2017) pointed out how persons with dementia and their caretakers appreciated museums as supportive and welcoming spaces. Carswell et al., (2021) continued on the power of art activities to transform clinical environments into more human and creative settings for patients receiving hemodialysis. Our findings reverberate these arguments, and suggest a welcoming environment as an important underlying mechanism for a diverse group of older adults who engage with different art forms in a variety of settings.

Anderson’s (2009) notion of affective atmospheres helps to understand the role of this environment. He points to atmospheres’ ambivalent status, being a “real phenomenon” and at the same time “not necessarily sensible” (Anderson’s (2009) p. 78). Atmospheres can thus seem vague and hard to rationalize, while at the same time older adults and artists experienced something which is real. They experienced singular affective qualities that express a world of for example, acceptance, trust or freedom, which enabled participants to connect, flow, relax and feel supported in their wellbeing. Anderson further emphasizes the unfinished nature of atmospheres, constantly emerging and transforming, being “reworked through (new) lived experiences” (Anderson’s (2009) p. 79). Sometimes older adults valued group work, while other times – or for other participants – feeling welcome and acceptance stood central. Such ephemerality appears hard to grasp. Therefore, we argue for the importance to consider the context wherein atmospheres and underlying mechanisms emerged, because it offers concrete entry points for analysis.

Regarding that context (C), Bennett’s (2009) concept of agentic assemblages balances concrete objects of analysis with coherence and conceptual flexibility. Her idea is rooted in Deleuze and Guattari’s work on the assemblage “as a mode of ordering heterogeneous entities so that they work together for a certain time” (Müller, 2015, p. 28). Conceptualized as socio-material gatherings wherein (non-)human members relate, assemblages produce new behaviors, expressions and realities. Built on Spinoza’s conative bodies, Bennett (2009. 23) continues how members’ interactions result in an emergent agency of the assemblage. This agency is not governed by one central head and is furthermore different from the sum of its individual members’ agencies. It is a distributed agency. When approaching participatory art activities as agentic assemblages, it is in the specific interactions and connections between participants, artists, materials, the art form and continuity of activities that intertwined and situated underlying mechanisms and outcomes emerged. Of course, the outcomes are grounded in older adults’ lived experiences, competences and needs. Artists with their creative and social competencies were as crucial, in guiding participants’ processes of growth (Possibility) and states of flow (Transformation). Especially social skills were important in working with older adults on the intersection of arts and health. Empathy enabled artists to offer emotional support when needed (Humanity). Combined with creative skills – and an appropriate art form such as contemporary dance – it allowed artists to work with older adults’ physical and cognitive capabilities (Possibility). In short, creative and social skills contributed to participants feeling stimulated, welcomed and supported. Together, participants and artists co-created context-specific affective atmospheres that reflected acceptance of identities and the freedom to express (Involvement). But also material elements contributed to a welcoming environment. The art studios’ interior for example, – often in strong contrast with the daily context – with its artistic materials, coffee, tea and music further added to the atmosphere. On top, also the structural implementation of activities can be interpreted as a member of the assemblage. Workshops with continuity in artists, participants, location and frequency of activities offered valuable day and week structure that supported participants’ wellbeing (Humanity). Interruptions of activities due to COVID-19 lockdowns, foregrounded this. It points to a temporal dimension that is not included in Cousins et al.,’s (2020) 10 context dimensions. Agentic assemblages on the other hand, entail temporality since they are open ended collectives, always gathering, dispersing, transforming and (dis)assembling over time (Bennett, 2009; Müller, 2015).

Future research on underlying mechanisms could benefit from including this temporal dimension in case-based longitudinal studies, to investigate how the frequency of workshops – daily, (bi)weekly – and duration of the art project – project based versus continuous – possibly influences (sustained) impact. It can also focus on the open-ended nature of agentic assemblages how over time (dis)continuity of specific members impacts distributed agencies. Also, incorporating participants’ gender identity could deepen our understanding of what works for whom, as most of the participants and interviewees were women. Future research could continue to integrate Cousins et al., (2020) taxonomy with affective atmospheres and agentic assemblages by applying them on different art forms, care settings, care needs, and generations. Herein, analyses of underlying mechanisms might benefit from a specific focus on members of the assemblage, affective atmospheres and the principle Involvement as they turned out to be key components.

### Strengths and limitations

We demonstrated the applicability of Cousins et al.,’s (2020) taxonomy to older adults with diverse care needs, a variety of art forms, (care) settings,, and enriched the taxonomy by aid of agentic atmospheres and agentic assemblages. Unfortunately, all interviews with older adults were conducted by telephone because data collection took place at the start of the COVID-19 pandemic. For that reason, this data mostly represents independent, verbally strong and cognitively abled persons, while persons with dementia or residents in nursing homes are less represented. Another limitation is the selection of participatory art projects in the more extensive nationwide participatory action research project. This procedure was done by the funding agencies and might have shaped the findings of this article.

## Conclusion

The aim of this study was to investigate the mechanisms that underlie potential benefits of active art engagement in terms of older adults’ wellbeing. First of all, the study adds to existing evidence that participatory arts can contribute to social connections, growth on a personal and artistic level and support in psychosocial wellbeing. Secondly, we showed that Cousins et al.,’s (2020) taxonomy on arts interventions, worked well when applying to older adults with a spectrum of care needs and various art forms. The principle Involvement with its welcoming environment appeared a consistent underlying mechanism for other principles and outcomes. This discloses the important social role participatory art can have for older adults. On an affective conceptual level, future research in this field could benefit from Anderson’s (2009) notion of affective atmospheres to deepen our understanding of welcoming environments in art projects. On a more rationalizing level, the concept of agentic assemblages Bennett, 2009 offers a suitable alternative to the rather static “context” dimensions in Cousins et al.,’s (2020) taxonomy. It enables us to unpack how socio-material gatherings co-create the right contextual conditions for outcomes and underlying mechanisms to emerge. This might be crucial for attuning to the contingent nature of art practices. It furthermore offers concrete objects for analysis, as well as opportunities for improving artistic practices.
